# Temporal Trends and Demographic and Geographic Disparities in Stroke Mortality Among US Adults Aged 25-64 Years (1999-2020): A CDC Wide-Ranging Online Data for Epidemiologic Research (WONDER) Analysis

**DOI:** 10.7759/cureus.113934

**Published:** 2026-08-04

**Authors:** Ronit Farahmandian, Mariam Kananyan, Jorge E Alonso, Naomi Saro-Naenwi, Tolulope Ajibade, Purvi Kaurani, Vamsi Krishna Gudapati, E. Lucano Gómez-Sauceda, Cleve Clarke, Srihitha Arra, Bashir Imam, Anchal R Gupta, Yahya Makkieh

**Affiliations:** 1 Internal Medicine, California University of Science and Medicine, Los Angeles, USA; 2 Faculty of Medicine, Yerevan State Medical University, Armenia, ARM; 3 Neurosurgery, Universidad Catolica de Santiago de Guayaquil, Guayaquil, ECU; 4 Department of General Medicine, Ministry of Health, Mater Dei Hospital, Malta, MLT; 5 General Practice, Royal College of General Practitioners, Stoke-on-Trent, GBR; 6 Sports and Exercise Medicine, University of South Wales, Wales, GBR; 7 Neurology, DY Patil University School of Medicine, Navi Mumbai, IND; 8 Faculty of Medicine, BGS Global Institute of Medical Sciences, Bangalore, IND; 9 Internal Medicine, Universidad Autónoma de Nuevo León, Nuevo Leon, MEX; 10 Faculty of Medicine, University of Technology, Kingston, JAM; 11 General Internal Medicine, Gandhi Medical College and Hospital, Secunderabad, IND; 12 Pediatrics, University of Pittsburgh Medical Center, Coudersport, USA; 13 Internal Medicine, Royal Shrewsbury Hospital, Shrewsbury, GBR; 14 Faculty of Medicine, Beirut Arab University, Beirut, LBN

**Keywords:** age-adjusted mortality rate, cdc wonder, geographic disparities, joinpoint regression, racial disparities, sex disparities, stroke mortality, temporal trends, urban–rural differences

## Abstract

Background: Stroke remains a major contributor to mortality in the United States. Understanding long-term temporal changes and demographic and geographic disparities in stroke mortality is essential for identifying high-risk populations and guiding targeted prevention strategies.

Methods: This retrospective observational study analyzed stroke mortality among US adults aged 25-64 years from 1999 to 2020 using the Centers for Disease Control and Prevention Wide-ranging Online Data for Epidemiologic Research (CDC WONDER) database. Deaths with stroke recorded as the underlying cause were identified using International Classification of Diseases, 10th Revision (ICD-10) codes I60-I64 and were analyzed collectively as overall stroke mortality. Age-adjusted mortality rates per 100,000 population were assessed overall and stratified by sex, race, US Census region, and urbanization level. Joinpoint regression was used to identify temporal changes and estimate the annual percent change (APC) for each temporal segment and the average annual percent change (AAPC) across the complete study period.

Results: A total of 382,253 stroke deaths were identified. The overall age-adjusted mortality rate (AAMR) decreased significantly from 1999 to 2012 (APC, -2.41%; 95% CI, -2.77 to -2.21), remained statistically unchanged from 2012 to 2018, and increased significantly from 2018 to 2020 (APC, 4.36%; 95% CI, 1.51 to 6.06). The full-period AAPC was -1.23% (95% CI, -1.42 to -1.11). Men had higher AAMRs than women and experienced a significant increase during 2018-2020. Black or African American adults had the highest race-specific AAMR, the South had the highest regional AAMR, and noncore counties had the highest urbanization-specific AAMR. Full-period AAPCs were significantly negative across all examined subgroups; however, significant later-period increases occurred among men, American Indian or Alaska Native adults, White adults, residents of the West, large fringe metropolitan counties, and noncore counties.

Conclusion: Premature stroke mortality declined substantially over the complete study period, but progress was uneven across demographic and geographic groups. Persistently elevated mortality and recent reversals in selected populations highlight the need for targeted prevention and continued surveillance.

## Introduction

Stroke remains a major cause of disability and death in the United States and has been ranked among the leading causes of death nationally [1,2]. In 2021, approximately 410,000 incident stroke cases were estimated [3]. Despite advances in stroke treatment, many survivors experience long-term physical, psychological, and cognitive sequelae. Consequently, prevention remains central to reducing stroke-related morbidity and mortality, with key strategies including hypertension control, smoking cessation, diabetes management, and lifestyle modification.

Although stroke mortality has generally declined in the United States, improvements have not been uniformly distributed across population groups. Important differences persist by sex, race, geographic region, and urbanization level. Rural-urban differences in socioeconomic vulnerability, healthcare access, and cardiovascular risk factors may contribute to heterogeneous stroke outcomes, but these patterns remain insufficiently characterized in national mortality analyses [4-6].

The Centers for Disease Control and Prevention Wide-Ranging Online Data for Epidemiologic Research (CDC WONDER) database provides national mortality and population data derived from US death certificates. In this study, we evaluated temporal trends in overall stroke mortality among adults aged 25-64 years in the United States from 1999 through 2020 using ICD-10 codes I60-I64. Joinpoint regression was used to identify changes in mortality trends and estimate annual percentage changes and average annual percentage changes [7-9]. Analyses were stratified by sex, race, US Census region, and urbanization level to identify populations that may benefit from targeted stroke-prevention and public health interventions.

## Materials and methods

Death record identification and eligibility

Mortality data were obtained from the CDC WONDER Multiple Cause of Death database from January 1999 through December 2020. Deaths with stroke recorded as the underlying cause were identified using International Classification of Diseases, 10th Revision (ICD-10) codes I60-I64 [10]. These codes included subarachnoid hemorrhage (I60), intracerebral hemorrhage (I61), other nontraumatic intracranial hemorrhage (I62), cerebral infarction (I63), and stroke not specified as hemorrhage or infarction (I64). All five categories were pooled and analyzed collectively as overall stroke mortality. Death records were included when one of these codes was recorded as the underlying cause of death. Because the study used publicly available, de-identified aggregate mortality data, institutional review board approval and informed consent were not required. The study was reported in accordance with the Strengthening the Reporting of Observational Studies in Epidemiology guidelines. The analysis was restricted to decedents aged 25-64 years, corresponding to the 25-34-, 35-44-, 45-54-, and 55-64-year age groups.

Data abstraction

Demographic and geographic variables included sex, race, US Census region, and urbanization level. Race categories included White, Black or African American, American Indian or Alaska Native, and Asian or Pacific Islander, according to the classifications available in CDC WONDER. Hispanic origin was not analyzed separately; therefore, the racial categories included persons of both Hispanic and non-Hispanic origin. US Census regions were categorized as Northeast, Midwest, South, and West. Urbanization level was classified using the 2013 National Center for Health Statistics Urban-Rural Classification Scheme for Counties. Counties were categorized as large central metropolitan, large fringe metropolitan, medium metropolitan, small metropolitan, micropolitan, or noncore according to the decedent’s county of legal residence.

Statistical analysis

Age-adjusted mortality rates (AAMRs) per 100,000 population, corresponding 95% confidence intervals, and standard errors were obtained directly from CDC WONDER and standardized to the 2000 US standard population. Annual and full-period AAMRs were assessed overall and stratified by sex, race, US Census region, and urbanization level. Temporal trends were evaluated using the Joinpoint Regression Program, version 6.0.1 [7]. Log-linear models were fitted to annual AAMRs using standard errors provided by CDC WONDER and an uncorrelated-error model. Grid search was used, with a minimum of zero and a maximum of four joinpoints. Model selection was performed using Monte Carlo permutation testing with 4,499 permutations and an overall significance level of 0.05 [8]. Annual percentage changes and corresponding 95% empirical-quantile confidence intervals were calculated for each identified temporal segment. Average annual percentage changes and corresponding 95% empirical-quantile confidence intervals were calculated for the complete study period from 1999 through 2020 [9]. Empirical-quantile confidence intervals for the final selected models were generated using 5,001 resamples.

All statistical tests were two-sided, and a p-value of less than 0.05 was considered statistically significant. A trend was described as significantly increasing or decreasing only when its 95% confidence interval excluded zero and its p-value was less than 0.05. Trends that did not meet these criteria were described as showing no statistically significant change.

## Results

From 1999 through 2020, 382,253 deaths with stroke recorded as the underlying cause were identified among US adults aged 25-64 years using ICD-10 codes I60-I64. The annual number of deaths ranged from 16,794 in 1999 to 18,938 in 2020. The overall AAMR decreased from 11.80 per 100,000 population in 1999 to 8.54 in 2018, before increasing to 8.58 in 2019 and 9.42 in 2020. Joinpoint regression identified changes in trend in 2012 and 2018. The AAMR decreased significantly from 1999 to 2012, with an annual percent change (APC) of -2.41% per year (95% CI, -2.77 to -2.21; p < 0.001), followed by no statistically significant change from 2012 to 2018 (APC, -0.48%; 95% CI, -1.83 to 0.37; p = 0.177). From 2018 to 2020, the AAMR increased significantly by 4.36% per year (95% CI, 1.51 to 6.06; p < 0.001). The full-period average APC (AAPC) was -1.23% (95% CI, -1.42 to -1.11; p < 0.001).

Sex

Among the 382,253 deaths identified during the study period, 214,252 occurred among men and 168,001 occurred among women, representing 56.05% and 43.95% of deaths, respectively. Across the complete study period, the AAMR was 11.0 per 100,000 population among men and 8.3 per 100,000 among women.

Joinpoint regression identified one joinpoint among women in 2013. The AAMR decreased significantly from 1999 to 2013, with an APC of -2.89% per year (95% CI, -3.16 to -2.67; p < 0.001). From 2013 to 2020, there was no statistically significant change in the AAMR (APC, 0.15%; 95% CI, -0.51 to 1.07; p = 0.593). The full-period AAPC among women was -1.89% (95% CI, -2.05 to -1.74; p < 0.001).

Among men, Joinpoint regression identified changes in trend in 2011 and 2018. The AAMR decreased significantly from 1999 to 2011 (APC, -2.10%; 95% CI, -3.05 to -1.75; p = 0.018), followed by no statistically significant change from 2011 to 2018 (APC, -0.45%; 95% CI, -1.68 to 0.83; p = 0.296). From 2018 to 2020, the AAMR increased significantly by 5.79% per year (95% CI, 1.72 to 7.96; p = 0.001). The full-period AAPC among men was -0.82% (95% CI, -1.10 to -0.66; p < 0.001).

**Figure 1:**
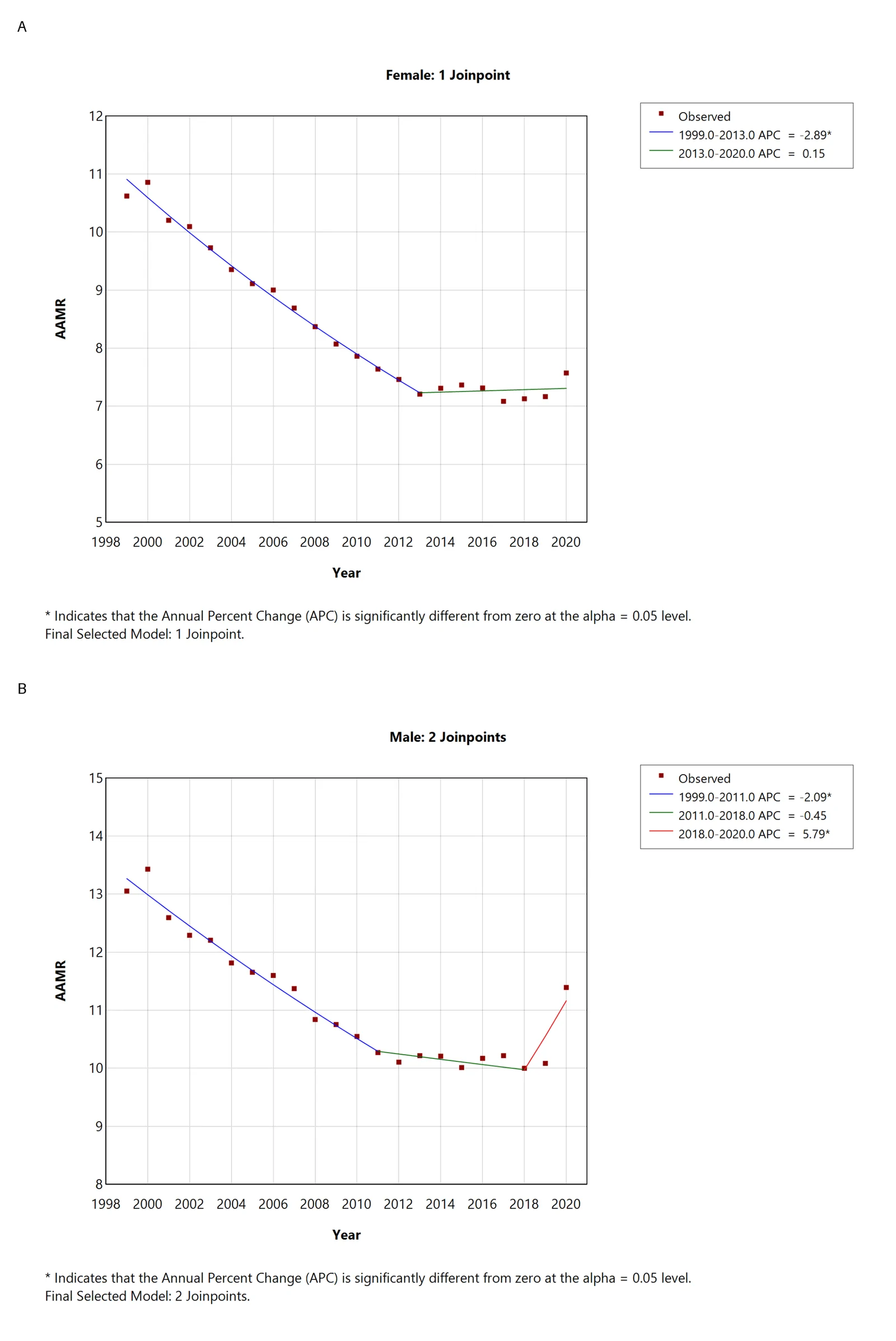
Joinpoint regression of age-adjusted stroke mortality trends by sex among US adults aged 25-64 years, 1999-2020 Panel A presents the mortality trend among women, and Panel B presents the trend among men. Squares represent observed annual age-adjusted mortality rates per 100,000 population, and fitted lines represent the final Joinpoint regression models. Among women, mortality decreased significantly from 1999 to 2013, followed by no statistically significant change from 2013 to 2020. Among men, mortality decreased significantly from 1999 to 2011, showed no statistically significant change from 2011 to 2018, and increased significantly from 2018 to 2020. An asterisk indicates an APC significantly different from zero at the 0.05 level. Y-axis scales vary across panels to improve visualization; absolute mortality levels should therefore be compared using the reported AAMRs rather than the visual height of the curves.

Men had higher AAMRs than women throughout the study period. Although both groups experienced significant overall declines from 1999 to 2020, the decline was numerically smaller among men, and a significant increase was identified among men during 2018-2020 (Figure 1).

Race

Of the 382,253 stroke deaths identified during the study period, 260,778 occurred among White adults, 101,430 among Black or African American adults, 16,616 among Asian or Pacific Islander adults, and 3,429 among American Indian or Alaska Native adults, representing 68.22%, 26.53%, 4.35%, and 0.90% of all deaths, respectively. Across the complete study period, the age-adjusted mortality rate was highest among Black or African American adults at 21.34 per 100,000 population, followed by Asian or Pacific Islander adults at 8.19, American Indian or Alaska Native adults at 8.04, and White adults at 7.98 per 100,000.

Among American Indian or Alaska Native adults, Joinpoint regression identified one joinpoint in 2018. The AAMR decreased significantly from 1999 to 2018, with an APC of -3.09% per year (95% CI, -4.58 to -2.28; p = 0.008), followed by a significant increase from 2018 to 2020 (APC, 20.06%; 95% CI, 0.65 to 30.50; p = 0.042). The full-period AAPC was -1.10% (95% CI, -2.40 to -0.31; p = 0.006).

Among Asian or Pacific Islander adults, one joinpoint was identified in 2008. The AAMR decreased significantly from 1999 to 2008 (APC, -4.41%; 95% CI, -6.94 to -3.27; p < 0.001). From 2008 to 2020, there was no statistically significant change in the AAMR (APC, -1.23%; 95% CI, -1.92 to 0.10; p = 0.058). The full-period AAPC was -2.61% (95% CI, -3.03 to -2.17; p < 0.001). Among Black or African American adults, joinpoints were identified in 2003, 2012, and 2018. There was no statistically significant change in the AAMR from 1999 to 2003 (APC, -85%; 95% CI, -3.15 to 1.69; p = 0.132), decreased significantly from 2003 to 2012 (APC, -4.16%; 95% CI, -6.37 to -3.71; p = 0.001), and continued to decrease significantly from 2012 to 2018 (APC, -2.08%; 95% CI, -3.81 to -0.53; p = 0.015). From 2018 to 2020, there was no statistically significant change in the AAMR (APC, 4.00%; 95% CI, -0.33 to 6.54; p = 0.071). The full-period AAPC was -2.38% (95% CI, -2.68 to -2.09; p < 0.001).

Among White adults, one joinpoint was identified in 2012. The AAMR decreased significantly from 1999 to 2012 (APC, -2.21%; 95% CI, -2.57 to -1.92; p < 0.001), followed by a significant increase from 2012 to 2020 (APC, 0.79%; 95% CI, 0.19 to 1.60; p = 0.013). The full-period AAPC was -1.08% (95% CI, -1.25 to -0.90; p < 0.001).

Although all four racial groups experienced significant overall reductions in AAMR during 1999-2020, Black or African American adults consistently had the highest mortality rates. Recent trends differed across groups, including a significant increase among American Indian or Alaska Native adults from 2018 to 2020 and a smaller but significant increase among White adults from 2012 to 2020.

**Figure 2:**
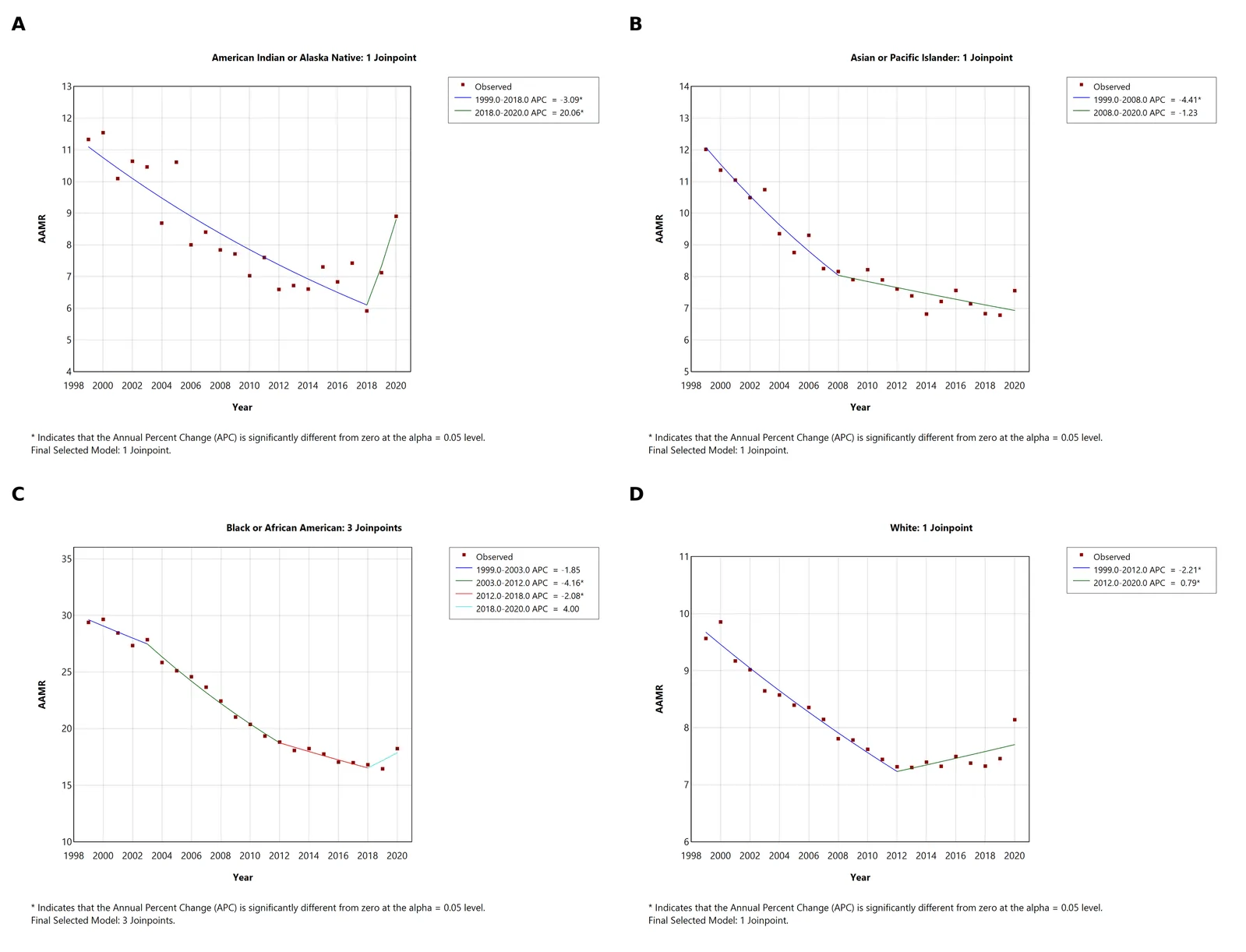
Joinpoint regression of age-adjusted stroke mortality trends by race among US adults aged 25-64 years, 1999-2020. Panels A–D present mortality trends among American Indian or Alaska Native, Asian or Pacific Islander, Black or African American, and White adults, respectively. Squares represent observed annual age-adjusted mortality rates per 100,000 population, and fitted lines represent the final selected Joinpoint regression models. An asterisk indicates that the annual percentage change was significantly different from zero at the 0.05 level. Y-axis scales vary across panels to improve visualization; absolute mortality levels should therefore be compared using the reported AAMRs rather than the visual height of the curves.

Data are presented in Figure 2.

Regional mortality patterns

**Figure 3:**
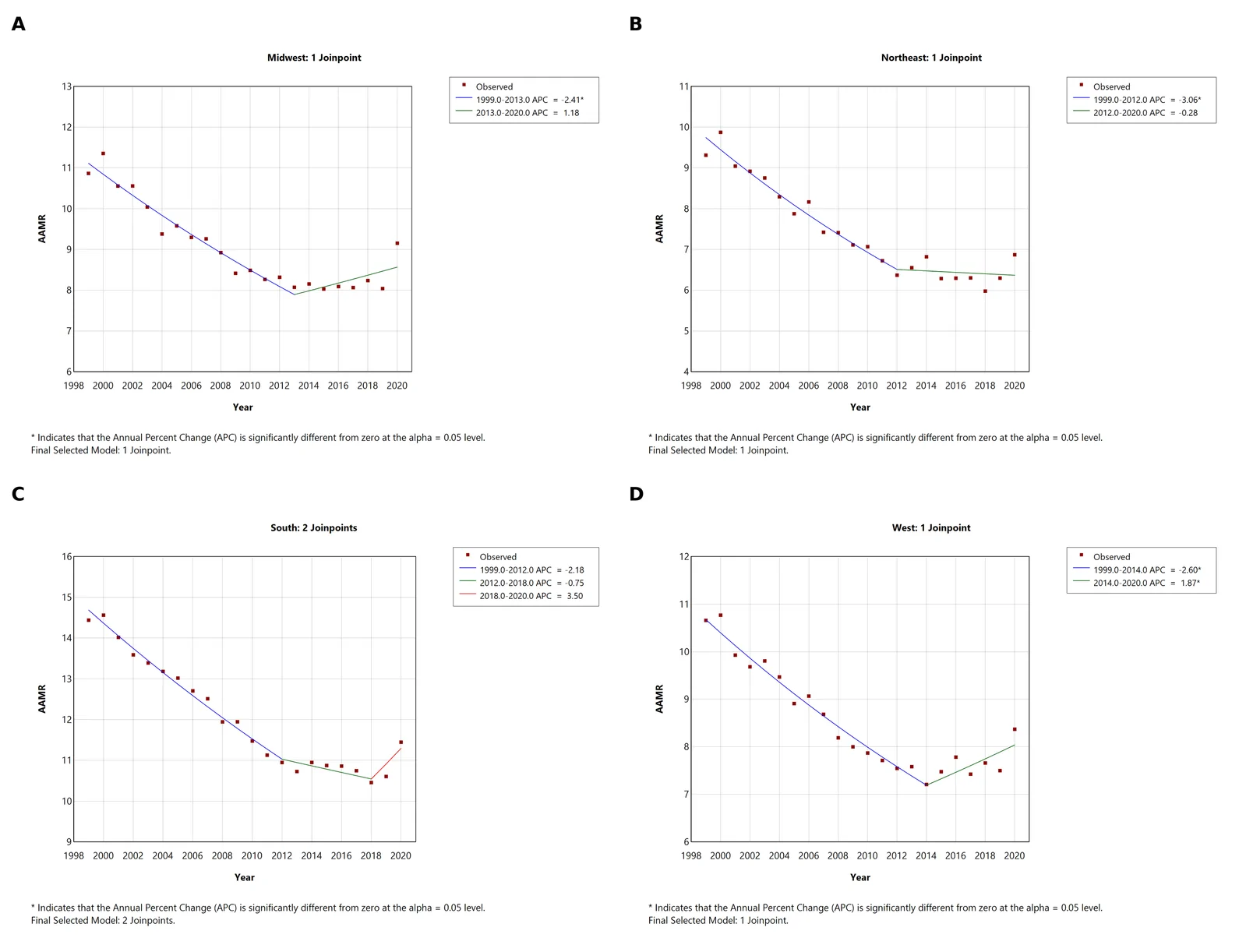
Joinpoint regression of age-adjusted stroke mortality trends by US Census region among adults aged 25-64 years, 1999-2020. Panels A–D present mortality trends in the Midwest, Northeast, South, and West, respectively. Squares represent observed annual age-adjusted mortality rates per 100,000 population, and fitted lines represent the final selected Joinpoint regression models. An asterisk indicates that the annual percentage change was significantly different from zero at the 0.05 level. Y-axis scales vary across panels to improve visualization; absolute mortality levels should therefore be compared using the reported AAMRs rather than the visual height of the curves.

The analysis revealed distinct mortality patterns across the Northeast, Midwest, South, and West, as shown in Figure 3.

Among the 382,253 stroke deaths identified from 1999 through 2020, 174,406 occurred in the South, 78,255 in the Midwest, 74,956 in the West, and 54,636 in the Northeast, representing 45.63%, 20.47%, 19.61%, and 14.29% of all deaths, respectively. Across the complete study period, the South had the highest age-adjusted mortality rate at 11.95 per 100,000 population (95% CI, 11.89-12.00), followed by the Midwest at 8.98 (95% CI, 8.92-9.05), the West at 8.38 (95% CI, 8.32-8.44), and the Northeast at 7.36 (95% CI, 7.29-7.42).

In the Midwest, Joinpoint regression identified one joinpoint in 2013. The AAMR decreased significantly from 1999 to 2013, with an APC of -2.41% per year (95% CI, -3.08 to -1.99; p < 0.001). From 2013 to 2020, there was no statistically significant change in the AAMR (APC, 1.18%; 95% CI, -0.06 to 3.87; p = 0.062). The full-period AAPC was -1.23% (95% CI, -1.55 to -0.93; p < 0.001).

In the Northeast, one joinpoint was identified in 2012. The AAMR decreased significantly from 1999 to 2012 (APC, -3.06%; 95% CI, -3.97 to -2.56; p < 0.001) and showed no statistically significant change from 2012 to 2020 (APC, -0.28%; 95% CI, -1.37 to 2.30; p = 0.741). The full-period AAPC was -2.01% (95% CI, -2.35 to -1.66; p < 0.001)

In the South, joinpoints were identified in 2012 and 2018. There was no statistically significant change in the AAMR from 1999 to 2012 (APC, -2.18%; 95% CI, -3.43 to 0.20; p = 0.052), from 2012 to 2018 (APC, -0.75%; 95% CI, -3.31 to 0.29; p = 0.097), or from 2018 to 2020 (APC, 3.50%; 95% CI, -0.12 to 5.57; p = 0.063). Despite the absence of statistically significant segment-specific changes, the full-period AAPC demonstrated a significant overall decline of -1.24% (95% CI, -1.51 to -1.07; p < 0.001).

In the West, one joinpoint was identified in 2014. The AAMR decreased significantly from 1999 to 2014 (APC, -2.60%; 95% CI, -3.19 to -2.15; p < 0.001), followed by a significant increase from 2014 to 2020 (APC, 1.87%; 95% CI, 0.29 to 6.15; p = 0.024). The full-period AAPC was -1.34% (95% CI, -1.67 to -1.04; p < 0.001).

Overall, all four Census regions experienced statistically significant declines over the complete study period. However, recent temporal patterns differed: the West increased significantly after 2014, whereas the later Midwest and South segments showed no statistically significant change.

Urbanization differences

**Figure 4:**
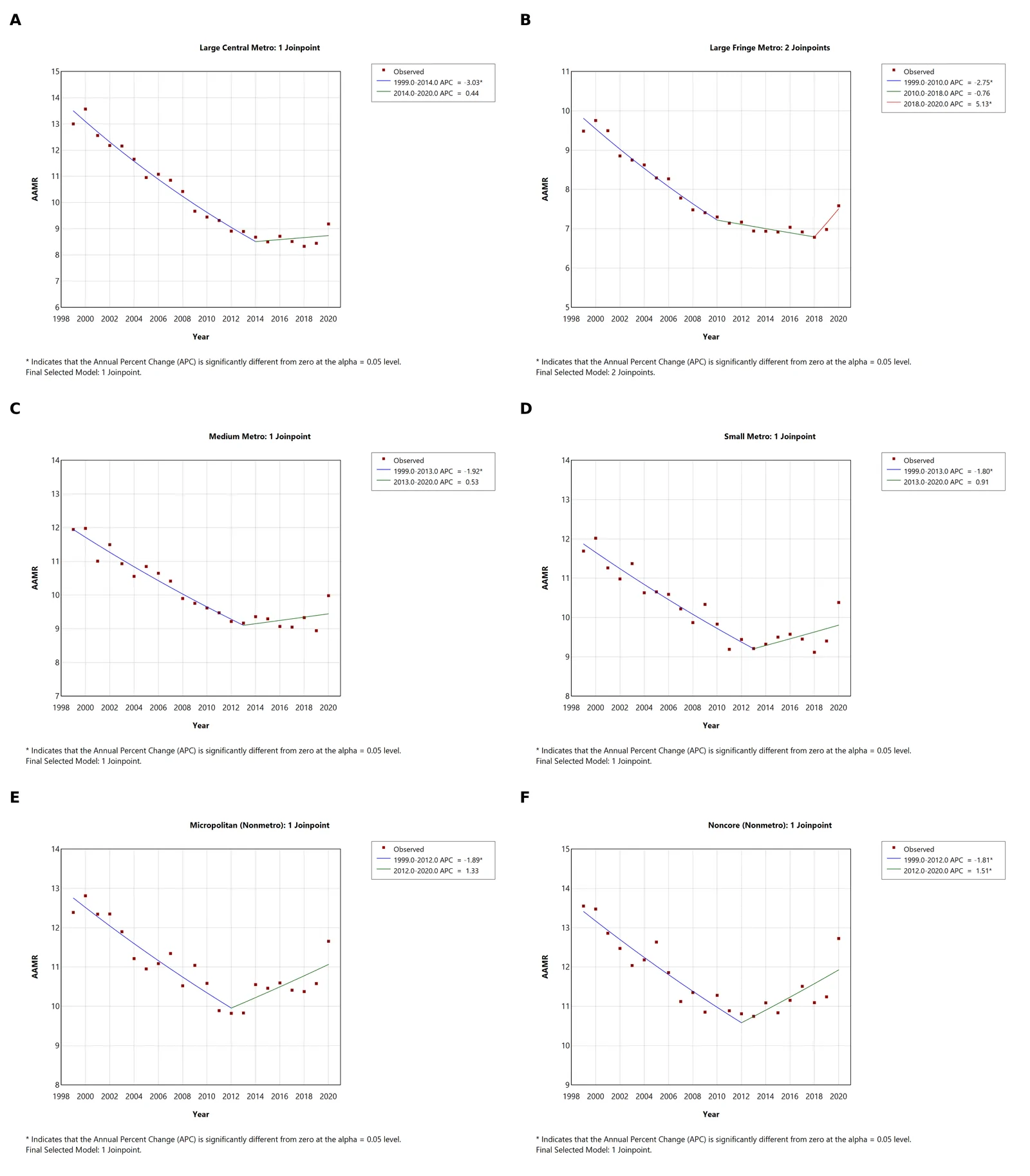
Joinpoint regression of age-adjusted stroke mortality trends by urbanization level among US adults aged 25-64 years, 1999-2020 Panels A–F present mortality trends in large central metropolitan, large fringe metropolitan, medium metropolitan, small metropolitan, micropolitan, and noncore counties, respectively. Squares represent observed annual age-adjusted mortality rates per 100,000 population, and fitted lines represent the final selected Joinpoint regression models. An asterisk indicates that the annual percentage change was significantly different from zero at the 0.05 level. Y-axis scales vary across panels to improve visualization; absolute mortality levels should therefore be compared using the reported AAMRs rather than the visual height of the curves.

The analysis of mortality by urbanization level revealed distinct patterns in AAMRs and temporal trends across the six categories, as shown in Figure 4.

Among the 382,253 stroke deaths identified during the study period, 116,570 occurred in large central metropolitan counties, 77,906 in large fringe metropolitan counties, 81,429 in medium metropolitan counties, 36,496 in small metropolitan counties, 39,155 in micropolitan counties, and 30,697 in noncore counties. These represented 30.50%, 20.38%, 21.30%, 9.55%, 10.24%, and 8.03% of all deaths, respectively. Across the complete study period, the pooled AAMR was highest in noncore counties at 11.65 per 100,000 population (95% CI, 11.52-11.79), followed by micropolitan counties at 10.98 (95% CI, 10.87-11.10), small metropolitan counties at 10.10 (95% CI, 9.99-10.20), large central metropolitan counties at 10.06 (95% CI, 10.00-10.12), medium metropolitan counties at 9.99 (95% CI, 9.92-10.06), and large fringe metropolitan counties at 7.69 (95% CI, 7.64-7.75).

In large central metropolitan counties, Joinpoint regression identified one joinpoint in 2014. The AAMR decreased significantly from 1999 to 2014, with an APC of -3.03% per year (95% CI, -3.93 to -2.61; p = 0.003), and showed no statistically significant change from 2014 to 2020 (APC, 0.44%; 95% CI, -1.36 to 5.88; p = 0.475). The full-period AAPC was -2.05% (95% CI, -2.45 to -1.75; p < 0.001).

In large fringe metropolitan counties, joinpoints were identified in 2010 and 2018. The AAMR decreased significantly from 1999 to 2010 (APC, -2.75%; 95% CI, -3.89 to -2.31; p = 0.014), showed no statistically significant change from 2010 to 2018 (APC, -0.76%; 95% CI, -2.15 to 0.36; p = 0.105), and increased significantly from 2018 to 2020 (APC, 5.13%; 95% CI, 0.67 to 7.35; p = 0.019). The full-period AAPC was -1.26% (95% CI, -1.57 to -1.08; p < 0.001).

In medium metropolitan counties, one joinpoint was identified in 2013. The AAMR decreased significantly from 1999 to 2013 (APC, -1.92%; 95% CI, -2.88 to -1.50; p = 0.002) and showed no statistically significant change from 2013 to 2020 (APC, 0.53%; 95% CI, -0.65 to 4.13; p = 0.305). The full-period AAPC was -1.11% (95% CI, -1.45 to -0.83; p < 0.001). In small metropolitan counties, one joinpoint was identified in 2013. The AAMR decreased significantly from 1999 to 2013 (APC, -1.80%; 95% CI, -2.46 to -1.40; p < 0.001), followed by no statistically significant change from 2013 to 2020 (APC, 0.91%; 95% CI, -0.19 to 3.48; p = 0.099). The full-period AAPC was -0.91% (95% CI, -1.20 to -0.62; p < 0.001).

In micropolitan counties, one joinpoint was identified in 2012. The AAMR decreased significantly from 1999 to 2012 (APC, -1.89%; 95% CI, -3.41 to -1.27; p = 0.002), followed by no statistically significant change from 2012 to 2020 (APC, 1.33%; 95% CI, -0.06 to 5.66; p = 0.060). The full-period AAPC was -0.67% (95% CI, -1.11 to -0.31; p = 0.001). In noncore counties, one joinpoint was identified in 2012. The AAMR decreased significantly from 1999 to 2012 (APC, -1.81%; 95% CI, -2.63 to -1.31; p < 0.001), followed by a significant increase from 2012 to 2020 (APC, 1.51%; 95% CI, 0.41 to 3.72; p = 0.007). The full-period AAPC was -0.56% (95% CI, -0.88 to -0.25; p < 0.001).

All six urbanization categories experienced statistically significant overall declines during the complete study period. Nevertheless, recent patterns differed, with significant increases in large fringe metropolitan counties after 2018 and noncore counties after 2012. Noncore counties had the highest pooled AAMR and the smallest full-period decline.

## Discussion

This national analysis identified 382,253 deaths with stroke recorded as the underlying cause among US adults aged 25-64 years from 1999 through 2020. Overall stroke mortality decreased significantly through 2012, showed no statistically significant change from 2012 to 2018, and increased significantly from 2018 to 2020. Despite this recent increase, the full-period AAPC remained significantly negative, indicating an overall long-term decline. Although the 2020 rate remained below the level observed in 1999, the subgroup analyses showed that improvements were not uniform. Significant full-period declines occurred across all examined sex, racial, regional, and urbanization groups, but several populations experienced persistently elevated mortality or recent unfavorable trends. Men had higher AAMRs than women, Black or African American adults had the highest race-specific AAMR, the South had the highest regional AAMR, and noncore counties had the highest urbanization-specific AAMR. Significant recent increases were also identified among men, American Indian or Alaska Native adults, White adults, residents of the West, residents of large fringe metropolitan counties, and residents of noncore counties.

The sex-stratified analysis demonstrated higher population-level stroke mortality among men throughout the study period. Mortality declined significantly among both sexes over the full period, but the reduction was smaller among men, who subsequently experienced a significant increase from 2018 to 2020. Women experienced a significant decline through 2013, followed by no statistically significant change. These findings should be interpreted as differences in population mortality rates rather than individual case fatality after stroke. Prior patient-level studies have reported complex sex differences in long-term outcomes after stroke, with the apparent association varying after adjustment for age, stroke severity, comorbidities, and other clinical factors [11-13]. Therefore, the higher male AAMR observed in the present study may reflect differences in stroke incidence, cardiovascular risk-factor burden, healthcare utilization, or competing population characteristics and does not independently demonstrate that men have worse survival after an incident stroke.

Marked racial disparities were also observed. Black or African American adults had a pooled AAMR of 21.34 per 100,000 population, substantially higher than the rates observed in the other racial groups. Despite this elevated burden, mortality declined significantly over the full study period, with the most pronounced reductions occurring between 2003 and 2012 and continuing, at a slower rate, through 2018. The increase observed from 2018 to 2020 was not statistically significant. White adults experienced a significant decline through 2012, followed by a smaller but significant increase through 2020. Asian or Pacific Islander adults experienced a pronounced decline through 2008, followed by no statistically significant change. Among American Indian or Alaska Native adults, the AAMR declined significantly through 2018 but increased sharply from 2018 to 2020. The latter estimate should be interpreted cautiously because it was based on a short two-year segment, involved a comparatively small number of deaths, and had a wide confidence interval. Nevertheless, the persistence of high mortality among Black or African American adults and the recent increase among American Indian or Alaska Native adults warrant continued surveillance. Prior research has documented racial differences in stroke mortality, access to acute stroke treatment, and stroke-related outcomes, which may reflect differences in structural conditions, healthcare access, prevention, and cardiovascular risk-factor exposure [14,15].

Geographic patterns further demonstrated that national improvements were uneven. The South accounted for the largest proportion of deaths and had the highest pooled regional AAMR, consistent with previous evidence of excess stroke mortality in the southern United States [14]. Although the South experienced a significant overall decline across 1999-2020, none of its individual Joinpoint segments reached statistical significance, and the final segment showed no statistically significant change after 2018. The Northeast experienced the largest full-period regional decline and remained statistically stable after 2012. The Midwest declined significantly through 2013, followed by no statistically significant change. The West declined significantly through 2014 but subsequently experienced a significant increase through 2020. These findings indicate that the longstanding geographic burden of stroke mortality cannot be characterized solely by a persistent South-versus-non-South difference; more recent unfavorable trends were also evident in western states.

The urbanization analysis showed that stroke mortality did not follow a completely linear urban-rural gradient across all six categories. Large fringe metropolitan counties had the lowest pooled AAMR, whereas noncore counties had the highest. Noncore counties also experienced the smallest full-period decline and a significant increase after 2012. Micropolitan and small metropolitan counties showed no statistically significant change after their respective joinpoints. Large fringe metropolitan counties experienced a significant increase from 2018 to 2020, whereas large central and medium metropolitan counties remained statistically stable after earlier declines. These patterns suggest that both rural areas and selected metropolitan populations may be vulnerable to recent reversals. Rural communities may face a greater burden of hypertension, diabetes, smoking, obesity, socioeconomic disadvantage, longer travel times, and limited access to preventive, emergency, and specialist stroke services [4-6]. However, these factors were not directly measured in the present analysis, and the observed associations should not be interpreted as causal.

The overall decline in stroke mortality is broadly consistent with national reports describing improvements in stroke prevention and treatment over previous decades [16-17]. Greater recognition and treatment of hypertension, expanded use of lipid-lowering and antithrombotic therapies, improved emergency stroke systems, and reductions in smoking may have contributed to these gains. At the same time, the slowing or reversal of mortality trends in selected groups may reflect persistent or worsening exposure to modifiable cardiovascular risk factors. Hypertension, diabetes, obesity, tobacco use, physical inactivity, and unhealthy dietary patterns remain central targets for primary stroke prevention, particularly among younger and middle-aged adults [18-19]. Because these clinical and behavioral variables were unavailable in CDC WONDER, the present study could not determine which factors accounted for the observed temporal changes.

The findings have several public health implications. First, national declines should not obscure the markedly elevated mortality observed among Black or African American adults, residents of the South, and residents of noncore counties. Second, the recent increases observed in several groups support continued monitoring rather than assuming that earlier improvements will persist. Third, prevention strategies should be adapted to the populations and settings experiencing the greatest burden. Such strategies may include improved hypertension detection and control, diabetes management, smoking cessation, obesity prevention, public recognition of stroke symptoms, timely emergency transport, and equitable access to primary prevention and acute stroke services. Interventions should also account for regional healthcare capacity, transportation barriers, insurance coverage, and other social determinants that may influence the prevention and treatment of stroke.

This study has several strengths. It used a national mortality database covering 22 years and included all US deaths meeting the predefined age and underlying-cause criteria. AAMRs were standardized to the 2000 US population, allowing comparisons across time and population groups. The use of Joinpoint regression permitted the identification of changes in trend that would not have been apparent from comparisons of isolated calendar years. The study also evaluated several demographic and geographic dimensions using a consistent analytic approach.

Several limitations should be considered. First, CDC WONDER data are derived from death certificates and are subject to coding errors and misclassification of the underlying cause of death. Race may also be misclassified on death certificates, particularly among American Indian or Alaska Native populations, which could affect estimates for smaller groups. Second, the study included only deaths for which stroke was recorded as the underlying cause and did not capture deaths in which stroke was listed solely as a contributing cause. Third, the database did not contain information on stroke severity, incident versus recurrent stroke, comorbidities, treatment, medication use, socioeconomic status, insurance coverage, or access to stroke centers. The analysis therefore cannot distinguish whether disparities resulted from differences in incidence, case fatality, risk-factor prevalence, healthcare access, or other factors.

Fourth, ICD-10 codes I60-I64 were pooled to evaluate overall stroke mortality. Consequently, the study could not determine whether trends differed between subarachnoid hemorrhage, intracerebral hemorrhage, cerebral infarction, other nontraumatic intracranial hemorrhage, and unspecified stroke. Fifth, Hispanic origin was not analyzed separately, and the racial categories included persons of both Hispanic and non-Hispanic origin. Sixth, the 2013 NCHS Urban-Rural Classification Scheme was applied throughout the 1999-2020 study period and may not reflect changes in county urbanization over time. Seventh, the ecological and aggregate nature of the data prevents causal or individual-level inference.

Finally, several recent trend segments included relatively few annual observations, particularly the 2018-2020 segments. Their APC estimates may therefore be sensitive to annual variation and should be interpreted alongside their confidence intervals and the underlying death counts. Multiple subgroup models were also evaluated without a study-wide adjustment for multiple comparisons. In addition, because the study ended in 2020, it cannot determine whether the increases observed near the end of the study period represented temporary fluctuations or sustained changes. Continued analyses incorporating subsequent years will be necessary to establish whether these unfavorable trends persisted.

Overall, premature stroke mortality declined substantially among US adults aged 25-64 years from 1999 through 2020, but the magnitude of improvement varied across demographic and geographic groups. Persistently elevated mortality among men, Black or African American adults, residents of the South, and residents of noncore counties, together with recent increases in selected populations, demonstrates that progress has been incomplete. Continued surveillance and targeted, equitable stroke-prevention strategies remain necessary to reduce these disparities.

## Conclusions

In this national CDC WONDER analysis, 382,253 deaths with stroke recorded as the underlying cause were identified among US adults aged 25-64 years from 1999 through 2020. Overall mortality decreased significantly from 1999 to 2012, remained statistically unchanged through 2018, and increased significantly from 2018 to 2020. Nevertheless, the full-period AAPC remained significantly negative. Improvements were uneven across demographic and geographic groups. Men, Black or African American adults, residents of the South, and residents of noncore counties had the highest AAMRs within their respective comparisons. Significant recent increases were identified among men, American Indian or Alaska Native adults, White adults, residents of the West, large fringe metropolitan counties, and noncore counties. These findings support continued surveillance and targeted stroke-prevention strategies for populations with persistently elevated mortality or recent unfavorable trends.
